# Radiomic Features of ^18^F-FDG PET in Hodgkin Lymphoma Are Predictive of Outcomes

**DOI:** 10.1155/2021/6347404

**Published:** 2021-11-22

**Authors:** Yeye Zhou, Yuchun Zhu, Zhiqiang Chen, Jihui Li, Shibiao Sang, Shengming Deng

**Affiliations:** ^1^Department of Nuclear Medicine, The First Affiliated Hospital of Soochow University, Suzhou, China; ^2^Department of Nuclear Medicine, First People's Hospital of Kunshan, Kunshan, China; ^3^Nuclear Medicine Laboratory of Mianyang Central Hospital, Mianyang 621099, China; ^4^State Key Laboratory of Radiation Medicine and Protection, Soochow University, Suzhou,215123, China

## Abstract

**Purpose:**

In the present study, we aimed to investigate whether the radiomic features of baseline ^18^F-FDG PET can predict the prognosis of Hodgkin lymphoma (HL).

**Methods:**

A total 65 HL patients (training cohort: *n* = 49; validation cohort: *n* = 16) were retrospectively enrolled in the present study. A total of 47 radiomic features were extracted from pretreatment PET images. The least absolute shrinkage and selection operator (LASSO) regression was used to select the most useful prognostic features in the training cohort. The distance between the two lesions that were the furthest apart (*D*_max_) was recorded. The receiver operating characteristic (ROC) curve, Kaplan–Meier method, and Cox proportional hazards model were used to assess the prognostic factors.

**Results:**

Long-zone high gray-level emphasis extracted from a gray-level zone-length matrix (LZHGE_GLZLM_) (HR = 9.007; *p*=0.044) and Dmax (HR = 3.641; *p*=0.048) were independently correlated with 2-year progression-free survival (PFS). A prognostic stratification model was established based on both risk predictors, which could distinguish three risk categories for PFS (*p*=0.0002). The 2-year PFS was 100.0%, 64.7%, and 33.3%, respectively.

**Conclusions:**

LZHGE_GLZLM_ and Dmax were independent prognostic factors for survival outcomes. Besides, we proposed a prognostic stratification model that could further improve the risk stratification of HL patients.

## 1. Introduction

Hodgkin lymphoma (HL) is a hematological malignancy, with an excellent prognosis for most patients [1]. However, a small number of patients still suffer from relapsed or refractory disease, and their prognosis is poor [2, 3]. The currently available prognostic indicators fail to identify high-risk patients [4, 5]. Therefore, it is urgently necessary to identify patients with a low or high risk of recurrence [6].

A combination of functional-metabolic and morphological imaging and 18F-fluorodeoxyglucose positron emission tomography/computed tomography (^18^F-FDG PET/CT) has become a standard imaging modality for HL patients [7–9]. Recently, a simple imaging feature measured on baseline ^18^F-FDG PET/CT can be useful in reflecting lesion dissemination of patients with lymphoma [10]. A high Dmax is associated with a poor prognosis [11].

**Figure 1:**
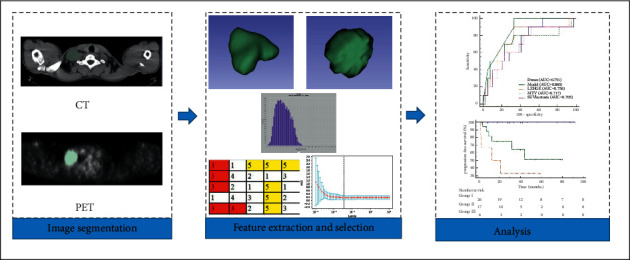
Workflow of the radiomics analysis. A 22-year-old man underwent ^18^F-FDG PET/CT for staging work-up of Hodgkin lymphoma (nodular sclerosis) with a maximum SUV of 10.95. The volume of interest (VOI) of the lymphoma lesion was manually delineated. 41% of SUV_max_ was applied as a threshold to optimize the VOI. The patient did not show progression and survived at the end of the 17-month follow-up period.

Radiomics is an emerging field that converts digital imaging data into a high-dimensional mineable feature space using high-throughput computing [12, 13]. By extracting a large number of quantitative features from tomographic images, radiomics has the potential to allow the assessment of tumor heterogeneity, which maybe correlated with clinical outcomes (Figure 1) [14–16]. Recent studies have reported the feasibility of radiomics in the prognosis of patients with various malignancies [15–18]. However, research using radiomics nomograms based on ^18^F-FDG PET for HL is relatively limited.

We, therefore, aimed to evaluate whether radiomic features derived from pretreatment ^18^F-FDG PET imaging could predict progression-free survival (PFS), alone or in combination with other parameters.

## 2. Materials and Methods

### 2.1. Patients

This retrospective study was approved by the institutional review board of the First Affiliated Hospital of Soochow University, and informed consent was waived. This study was carried out following the Declaration of Helsinki with a trial registration number of ChiCTR2100045957. All HL patients diagnosed from March 2013 to December 2020 were included in the present study. The inclusion criteria were set as follows: (1) histologically confirmed HL and (2) no chemo- or radiotherapy treatment before ^18^F-FDG PET/CT examination. Patients with other types of cancers or with incomplete clinical and imaging datasets were excluded.

A total of 65 patients (45 males and 20 females, mean age: 29 years, age range: 8–72 years) were randomly divided into the training (*n* = 49) and validation (*n* = 16) cohorts following a ratio of 7 : 3 [12, 19]. Clinicopathological data for each HL patient, including gender, age, B symptoms, level, Ann Arbor stage, bone marrow (BM) biopsy, bulky disease (>10 cm), *D*_max_, and PET/CT imaging data were acquired.

### 2.2. PET/CT Acquisition

All patients were asked not to eat for at least 6 h before the administration of ^18^F-FDG (4.07–5.55 MBq/kg). Blood glucose levels were less than 11 mmol/L. A whole-body scan was acquired at 60 ± 10 min after intravenous injection of ^18^F-FDG using an integrated PET/CT scanner (Discovery STE; General Electric Medical Systems, Milwaukee WI, USA). First, low-dose CT images were performed, with parameters as follows: 140 kV, 120 mA, a transaxial FOV of 70 cm, a pitch of 1.75, a rotation time of 0.8 s, and a slice thickness of 3.75 mm, followed by PET images, with 2-3 min per bed position and 7-8 bed position per patient.

### 2.3. Feature Extraction and Selection

The radiomic features were extracted from PET images using LIFEx freeware (v6.30 https://www.lifexsoft.org/) [20]. PET and CT images of the DICOM format were transferred to LIFEx freeware and automatically fused by the freeware. Areas with increased uptake of ^18^F-FDG on PET and abnormal density on CT were defined as lesions. The volume of interest (VOI) of the lymphoma lesion was manually delineated slice by slice using three-dimensional drawing tools by two experienced nuclear medicine physicians. Moreover, 41% of the maximum standardized uptake value (SUV_max_) was applied as a threshold to optimize the VOI [21]. Spatial resampling was 2 × 2 × 2 mm voxel size. Intensity discretization for PET data was processed with the number of gray levels of 64 bins and absolute scale bounds between 0 and 20 [22, 23]. After preprocessing, a total of 47 radiomic features were extracted from PET images, including conventional imaging parameters, histogram (HISTO), shape, gray-level co-occurrence matrix (GLCM), gray-level run-length matrix (GLRLM), neighborhood gray-level different matrix (NGLDM), and gray-level zone-length matrix (GLZLM) (Table 1).

A total of 15 patients were randomly selected to calculate the interobserver agreement of the feature extraction. The intraclass correlation coefficient (ICC) was used to determine the repeatability/reproducibility of features in our research, and ICC >0.75 was selected [24–26]. Subsequently, the least absolute shrinkage and selection operator (LASSO) COX regression model was used to select the most useful prognostic features with 10-fold cross validation for selecting the parameter Lambda in the training cohort [27, 28].

### 2.4. Treatment and Follow-Up

Patients were treated according to the institution's standard protocol. A total of 19 patients with early-stage disease (stage I and II without risk factors) were generally treated with an ABVD regimen (adriamycin, bleomycin, vinblastine, and dacarbazine). Moreover, 18 intermediate-stage patients generally received 4 to 6 cycles of ABVD, followed by involved-field radiotherapy. In addition, 31 advanced-stage patients (stage III and IV) were generally treated with 6 to 8 cycles of ABVD alone or a combination of chemotherapy and radiotherapy. Four patients received autologous stem cell transplantation after relapse. Patients were followed up by routine imaging methods (MRI, CT, or ^18^F-FDG PET/CT) every 3 months during the first 2 years and every 6 months thereafter. To allow earlier individual treatment, the PFS was set as the main endpoint [29].

### 2.5. Statistical Analysis

Statistical analyses were performed using SPSS software version 26.0 (SPSS Inc., Chicago, IL, USA) and python 3.0 (https://www.python.org). The differences in patients' characteristics between the training and validation cohorts were compared using the Chi-square test. The cutoff value of the radiomic features was defined by the receiver operating characteristic (ROC) curve according to Youden's index. The Kaplan–Meier method and log-rank test were used to estimate PFS. Multivariate analyses were performed using the Cox proportional hazards model. A *p* < 0.05 was considered statistically significant. The distances between all pairs of lesions (including both nodal and extranodal lesions) were calculated using the LIFEx software [20].

## 3. Results

### 3.1. Patient Characteristics

Table 2 summarizes the clinical and PET characteristics of patients in the training and validation cohorts. A total of 65 patients were enrolled in this study. Of these patients, 31 patients presented with nodular sclerosis, 14 patients presented with mixed cellularity, four patients presented as lymphocyte rich, two patients presented with lymphocyte depletion, and 14 patients presented with nodular lymphocyte-predominant subtypes. The relapse or progression of disease occurred in 14 patients (21.5%) with a median time of 11 months (range of 2–57 months). The median PFS was 40 months (range of 2–92 months). No significant differences were found between the two cohorts (*p* = 0.389–0.703).

### 3.2. Feature Selection in the Training Cohort

**Figure 2:**
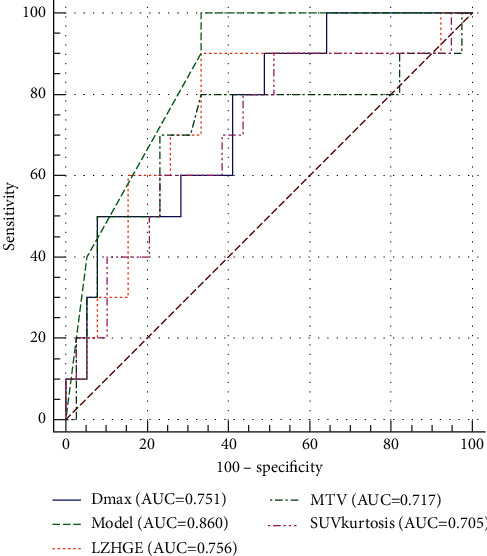
ROC curves and area under the curve (AUC) values of the radiomic features.

A total of 47 radiomic features were extracted in the training dataset. Based on the LASSO results, metabolic tumor volume (MTV), SUV kurtosis, and long-zone high gray-level emphasis extracted from the gray-level zone-length matrix (LZHGE_GLZLM_) were selected as potential prognostic factors for PFS. From ROC curves, the cutoff value of MTV was 135 cm^3^, SUV kurtosis was 5.6, and LZHGE_GLZLM_ was 3,200 (Figure 2). The ICC of the three radiomic features was 0.94, 0.80, and 0.84, respectively.

### 3.3. Univariate and Multivariate Analyses

Table 3 shows the results of univariate and multivariate analyses of the clinical parameters and PET variables that can discriminate different survival endpoints. The optimal cutoff value for *D*_max_ was 57.4 with an AUC of 0.751. In the univariate analysis, the BM biopsy, *D*_max_, MTV, SUV kurtosis, and LZHGE_GLZLM_ of radiomic features were associated with PFS. These variables were input into the multivariate Cox analysis. After multivariate analysis, LZHGE_GLZLM_ (HR = 9.007; *p*=0.044) and Dmax (HR = 3.641; *p*=0.048) remained prognostic factors for PFS.

**Figure 3:**
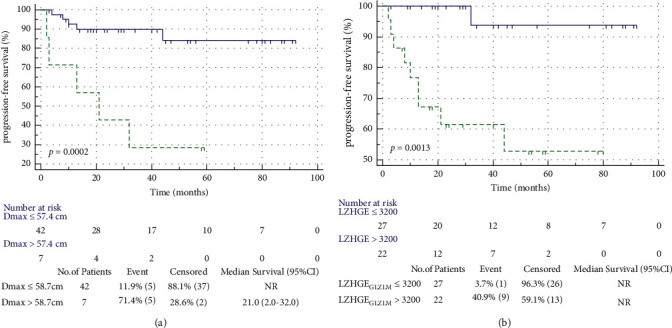
Kaplan–Meier survival analysis of PFS according to *D*_max_ (a) and LZHGE_GLZLM_ (b). NR, not reached.

High Dmax (>57.4 cm) and LZHGE_GLZLM_ (>3,200) were significantly associated with a shorter PFS (Figure 3). Patients with high Dmax had a 2-year PFS of 42.9%, whereas patients with low Dmax had a 2-year PFS of 90.5% (*p*=0.0002). Moreover, patients with high LZHGE_GLZLM_ had a 2-year PFS of 63.6%, whereas patients with low LZHGE_GLZLM_ had a 2-year PFS of 100.0% (*p*=0.0013).

### 3.4. Combination of Radiomic and Dissemination Features

**Figure 4:**
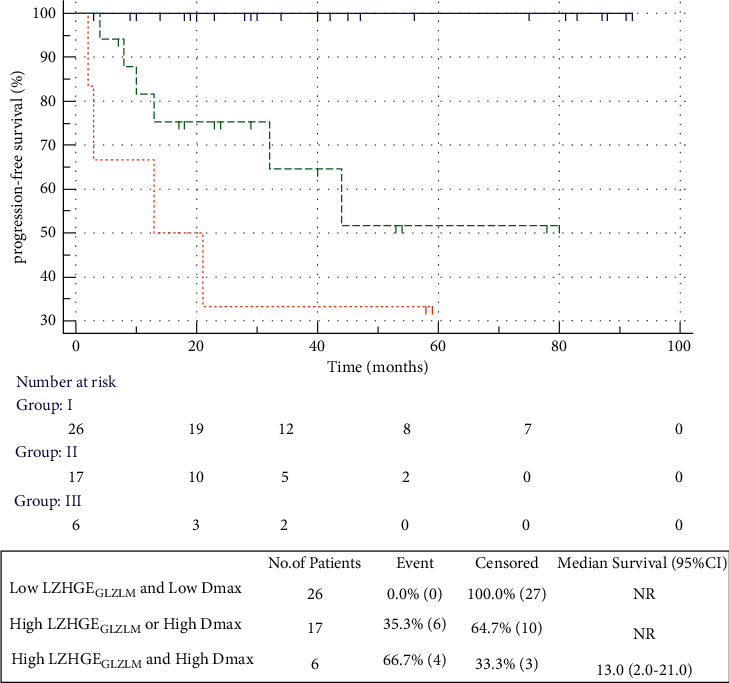
PFS according to LZHGE_GLZLM_ ≤ 3,200 and *D*_max_ ≤ 57.4 cm, LZHGE_GLZLM_ > 3,200 and *D*_max_ > 57.4 cm, and LZHGE_GLZLM_ > 3,200 and *D*_max_ > 58.7 cm. NR, not reached.

A prognostic stratification model was established based on the independent risk factors (Dmax andLZHGE_GLZLM_) presented in the multivariate analysis for PFS. Therefore, three risk categories could be significantly distinguished (*p*=0.0002) (Figure 4), including group I with no risk factors (*n* = 26); group II with one risk factor only (*n* = 17); and group III with two risk factors (*n* = 6), and the PFS of the abovementioned three groups was 100.0%, 64.7%, and 33.3% (*p*=0.0002), respectively. Comparison between group I and group II or between group I and group III revealed significantly different PFS (*p*=0.001, *p* < 0.0001, respectively), whereas comparison between group II and group III did not reach statistical significance (*p*=0.205).

## 4. Discussion

The present study demonstrated that ^18^F-FDG PET radiomic signature was useful for predicting survival outcomes in HL patients, and LZHGE_GLZLM_ and *D*_max_ were independent prognostic factors for PFS. Moreover, we established a prognostic stratification model based on two radiomic features, and HL patients were divided into three risk groups. The results indicated that PET radiomics might be helpful for prognostic evaluation of HL patients.

Intratumor heterogeneity is a recognized feature of malignancy, reflecting areas of high cell density, hypoxia, angiogenesis, and necrosis [30, 31]. It is a pivotal dimension associated with tumor aggressiveness and patient outcomes [32, 33]. Radiomics analysis of noninvasive imaging is a widely used approach to quantify intratumor heterogeneity [34]. Previous studies have shown that textural features can effectively predict treatment response and patient survival for various types of cancer [30, 35, 36]. Our results indicated that SUV kurtosis and LZHGE_GLZLM_ might improve the risk stratification in HL patients. Specifically, LZHGE_GLZLM_ was significantly related to PFS after multivariate analysis. Both radiomic features implied the measurement of intratumor heterogeneity. Kurtosis reflects the peak or flatness of an SUV intensity-volume histogram, and it is increased with higher heterogeneity [37]. LZHGE_GLZLM_ represents the distribution of the long homogeneous zones with high gray levels. A higher LZHGE_GLZLM_ is associated with a poor PFS.

At present, few studies have investigated the role of PET radiomics in predicting treatment outcomes in HL. Lue et al. [14] have found that SUV kurtosis is significantly related to PFS, and INU_GLRM_ is significantly associated with PFS and overall survival (OS). Another study has reported that wavelet HIR_GLRMPET and RLNU_GLRMCT are independent predictive factors for treatment response. The INU_GLRMPET and wavelet SRE_GLRMCT are associated with PFS, whereas ZSNU_GLSZMPET is a prognostic factor for OS [38]. Our findings were consistent with the abovementioned studies, indicating that PET radiomic features were useful for prognostic evaluation of HL patients.

Traditional PET metabolic parameters, such as MTV, have been proved to be significant prognostic indicators for the prognosis of HL patients [39, 40]. Parvez et al. have reported that the MTV can predict the response after therapy in 82 patients with aggressive B-cell lymphoma, while textural features cannot predict the treatment response, although several features are related to residual mass and outcomes [41]. However, several reports have demonstrated that the intratumor heterogeneity for survival prognostication is superior to traditional PET metabolic parameters [38, 42, 43]. Lue et al. have revealed that the pretreatment intensity nonuniformity of ^18^F-FDG PET is a promising prognostic indicator in HL patients and may outperform MTV [14]. In our present study, MTV was associated with PFS in the univariate analysis, while MTV did not retain the prognostic significance in the multivariate analysis. Many sources may cause these differences, such as small sample size, image segmentation, acquisition and reconstruction parameters, and feature extraction software [44]. Further investigations in a larger cohort population are required to validate our conclusions.

To the best of our knowledge, we, for the first time, predicted the survival outcomes of HL patients using the *D*_max_ feature. *D*_max_, which is the largest distance between all pairs of lesions, captures the spread of the disease. Recently, an analysis consisting of 95 patients with advanced-stage diffuse large B-cell lymphoma has reported that *D*_max_ is an independent predictor of PFS and OS. A high *D*_max_ was associated with an adverse prognosis, suggesting that the measurement of tumor dissemination was an essential biomarker for patients with lymphoma. The combination of PET radiomic features and *D*_max_ makes it possible to identify patients with a poor prognosis and guide clinicians to change treatment regimens [10]. In our present study, *D*_max_ was an independent prognostic factor of PFS, and the 2-year PFS in the high *D*_max_ and low *D*_max_ groups was 42.9% and 90.5%, respectively. Additionally, we established a prognostic stratification model based on Dmax and imaging features (LZHGE_GLZLM_) that predicted survival outcomes of HL patients. Indeed, patients with high *D*_max_ (>57.4 cm) and high LZHGE_GLZLM_ (>3,200) had a much worse prognosis compared with the other patients. The new model successfully improved patient risk stratification.

Repeatability and robustness are crucial in radiomics analysis [45]. In the present study, all ^18^F-FDG PET/CT images were realized in the same center using the same acquisition and reconstruction protocols. To reduce the impact of discretization values on robustness, a reliable discretization using a fixed size of bins was adopted [46]. Furthermore, our investigation of interobserver variability and LASSO logistic with 10-fold cross validation supported the robustness and prognostic power of the identified imaging features. Further external analysis of our results in a larger cohort is necessary and promotes the clinical application of radiomic features.

The present study has several limitations. First, this was a single-center retrospective study, and potential selection bias might exist. Second, the sample size was relatively small in the training cohort, particularly for the identification of available features in texture analysis. Besides, the interobserver variability could be affected by different image readers. Consequently, large-scale multicenter studies of the risk model are required to further verify its value.

## 5. Conclusions

Our results indicated the association between pretreatment ^18^F-FDG PET radiomic features and relapsed disease status in HL patients. Besides, a prognostic scoring system consisting of the Dmax and LZHGE_GLZLM_ could be useful to improve risk stratification, which might be beneficial for personalized treatment.

## Figures and Tables

**Table 1:** Radiomic parameters.

Index	Matrix	Parameter
Conventional		SUV_min_, SUV_mean_, SUV_max_, SUV_peak_, and SUV_Std_
Advanced indices		MTV and TLG
Histogram-derived parameters		Skewness, kurtosis, entropy, and energy
Shape-derived parameters		Sphericity and compacity
Texture features	GLCM	Homogeneity, energy, contrast, correlation, entropy, and dissimilarity
	GLRLM	SRE/LRE, LGRE/HGRE, SRLGE/SRHGE, LRLGE/LRHGE, GLNU/RLNU, and RP
	NGLDM	Coarseness, contrast, and busyness
	GLZLM	SZE, LZE, LGZE, HGZE, SZLGE, SZHGE, LZLGE, LZHGE, GLNU, ZLNU, and ZP

MTV: metabolic tumor volume; TLG: total lesion glycolysis; GLCM: gray-level co-occurrence matrix; GLRLM: gray-level run-length matrix; SRE/LRE: short/long-run emphasis; LGRE/HGRE: low/high gray-level run emphasis; SRLGE/SRHGE: short-run low/high gray-level emphasis; LRLGE/LRHGE: long-run low/high gray-level emphasis; GLNU/RLNU: gray-level nonuniformity/run-length nonuniformity; RP: run percentage; NGLDM: neighborhood gray-level difference matrix; GLZLM: gray-level zone-length matrix; SZE/LZE: short/long-zone emphasis; LGZE/HGZE: low/high gray-level zone emphasis; SZLGE/SZHGE: short-zone low/high gray-level emphasis; LZLGE/LZHGE: long-zone low/high gray-level emphasis; GLNU/ZLNU: gray-level nonuniformity or zone-length nonuniformity; ZP; zone percentage.

**Table 2:** Characteristics of the training and validation cohorts.

	Total (*n* = 65)	Training (*n* = 49)	Validation (*n* = 16)	*p*
Sex				0.596
Male	45 (69.2%)	34 (69.4%)	11 (68.8%)	
Female	20 (30.8%)	15 (30.6%)	5 (31.3%)	

Age, median (range)	29.0 (8–72)	29 (8–72)	30 (16–54)	0.703

Ann Arbor stage				0.585
I-II	36 (55.4%)	27 (55.1%)	9 (56.3%)	
III-IV	29 (44.6%)	22 (44.9%)	7 (43.8%)	

Bulky disease				0.678
>10 cm	9 (13.8%)	6 (12.2%)	3 (18.8%)	
<10 cm	56 (86.2%)	43 (87.8%)	13 (81.3%)	

BM				0.612
Yes	9 (13.8%)	7 (14.3%)	2 (12.5%)	
No	56 (86.2%)	42 (85.7%)	14 (87.5%)	

Extranodal sites				0.495
Yes	25 (38.5%)	20 (40.8%)	5 (31.3%)	
No	40 (61.5%)	29 (59.2%)	11 (68.8%)	

B symptoms				0.389
Yes	22 (33.8%)	18 (36.7%)	4 (25.0%)	
No	43 (66.2%)	31 (63.3%)	12 (75.0%)	

Chemotherapy with IFRT				0.565
Yes	4 (6.2%)	4 (8.2%)	0 (0.0%)	
No	61 (93.8%)	45 (91.8%)	16 (100.0%)	

LDH, lactate dehydrogenase, BM, bone marrow; IFRT, involved-field radiation therapy.

**Table 3:** Univariate and multivariate analyses for prognostic factors of PFS.

	Features	*p* value	HR (95% CI)
*Univariate analysis*			
Clinical parameters	Gender	0.1726	2.579 (0.6609–10.06)
	Age (>30)	0.2104	0.4514 (0.1300–1.567)
	Ann Arbor stage	0.0590	3.394 (0.9548–12.07)
	Extranodal sites	0.1788	2.398 (0.6700–8.581)
	B symptoms	0.2791	2.067 (0.5550–7.698)
	BM	0.0213^*∗*^	9.985 (1.363–46.78)
	Bulky disease (>10 cm)	0.2886	3.078 (0.3859–24.55)
	*D*_max_	0.0002^*∗*^	34.78 (5.206–232.4)
	Chemotherapy with IFRT	0.0908	8.589 (0.7105–103.8)
PET variables	SUVmax	0.0723	10.52 (0.8081–137.0)
	MTV	0.0016^*∗*^	9.811 (2.371–40.59)
	SUV kurtosis	0.0316^*∗*^	3.961 (1.129–13.90)
	LZHGE_GLZLM_	0.0013^*∗*^	8.036 (2.258–28.60)

*Multivariable analysis*			
	BM	0.086	—
	*D*_max_	0.048^*∗*^	3.641 (1.011–13.110)
	MTV	0.618	—
	SUV kurtosis	0.243	—
	LZHGE_GLZLM_	0.044^*∗*^	9.007 (1.066–76.116)

^*∗*^Statistically significant. HR, hazard ratio; CI, confidence interval; LDH, lactate dehydrogenase; BM, bone marrow; *D*_max_, the distance between the two lesions that were the furthest apart; MTV, metabolic tumor volume; LZHGE, long-zone high gray-level emphasis; GLZLM, gray-level zone-length matrix.

## Data Availability

The patient data used to support the findings of this study are available from the corresponding author upon request.
